# Breast cancer tumor growth estimated through mammography screening data

**DOI:** 10.1186/bcr2092

**Published:** 2008-05-08

**Authors:** Harald Weedon-Fekjær, Bo H Lindqvist, Lars J Vatten, Odd O Aalen, Steinar Tretli

**Affiliations:** 1 Department of Etiological Research, Cancer Registry of Norway, Institute of Population-based Cancer Research, Montebello, N-0310 Oslo, Norway; 2 Department of Biostatistics, Institute of Basic Medical Sciences, University of Oslo, Norway; 3 Department of Mathematical Sciences, Norwegian University of Science and Technology, Trondheim, Norway; 4 Department of Public Health, Norwegian University of Science and Technology, Trondheim, Norway

## Abstract

**Introduction:**

Knowledge of tumor growth is important in the planning and evaluation of screening programs, clinical trials, and epidemiological studies. Studies of tumor growth rates in humans are usually based on small and selected samples. In the present study based on the Norwegian Breast Cancer Screening Program, tumor growth was estimated from a large population using a new estimating procedure/model.

**Methods:**

A likelihood-based estimating procedure was used, where both tumor growth and the screen test sensitivity were modeled as continuously increasing functions of tumor size. The method was applied to cancer incidence and tumor measurement data from 395,188 women aged 50 to 69 years.

**Results:**

Tumor growth varied considerably between subjects, with 5% of tumors taking less than 1.2 months to grow from 10 mm to 20 mm in diameter, and another 5% taking more than 6.3 years. The mean time a tumor needed to grow from 10 mm to 20 mm in diameter was estimated as 1.7 years, increasing with age. The screen test sensitivity was estimated to increase sharply with tumor size, rising from 26% at 5 mm to 91% at 10 mm. Compared with previously used Markov models for tumor progression, the applied model gave considerably higher model fit (85% increased predictive power) and provided estimates directly linked to tumor size.

**Conclusion:**

Screening data with tumor measurements can provide population-based estimates of tumor growth and screen test sensitivity directly linked to tumor size. There is a large variation in breast cancer tumor growth, with faster growth among younger women.

## Introduction

Mammography screening is now an established part of the health service in developed countries. There is, however, still an ongoing discussion related to optimizing mammography screening, including determining optimal time intervals between screenings and which age groups to invite. For these decisions, adequate estimates of breast cancer tumor growth and screening test sensitivity (STS) are crucial. In addition, better knowledge of tumor growth will benefit the evaluation of screening programs [1], as well as the interpretation of clinical trials and epidemiological studies. There are some observational studies of patients that were initially overlooked at earlier mammograms [2-4] or were refused treatment [2,3], but these studies are small and are probably influenced by length of time bias, since slow-growing tumors spend relatively longer times in preclinical stages visible on mammograms. To our knowledge, no large-scale population-based clinical observational studies of untreated cancers have therefore been performed as cancers are usually treated in populations with good cancer surveillance.

Tumor growth can also be indirectly observed as tumor progression, estimated from variations in cancer incidence in screening trials or programs. These studies [1] are usually analyzed using Markov models [5,6], where the mean time for a breast cancer tumor to growth from screening-detectable size to clinical detection without screening – the so-called mean sojourn time – and the STS are estimated. The Markov model, however, has no separate variable for individual variation, and the estimated variables are highly correlated with contributions from both the underlying biological processes and the given screening method. The estimated parameters therefore have no explicit relation to the biological process of tumor growth, and are often difficult to compare between different countries, as the STS is defined as 'the proportion of cancers detected at screening among screening detectable cancers', using the evaluated procedural as its own reference.

Tumor growth can be estimated by comparing tumor sizes from clinical-detected and screening-detected cases, but the applied statistical models only partly utilize these data. Chen and colleagues [7] used tumor size in a classical Markov model, and van Oortmarssen and colleagues [8] used tumor size in a simulation approach – but both studies only categorized tumor size into two or three groups. On the contrary, some clinical observation studies fully utilize tumor size measurements with tumor growth modeled as a continuous function of tumor size [2,9], but these studies of nontreated or overlooked cancers are small and the results may not be valid due to either selection bias or length of time bias.

The aim of the present study was to utilize modern computer power on data from a population-based screening program, with precise standardized measurements of tumor size, to reliably estimate tumor growth and STS.

## Materials and methods

### Setting: data

In 1995 the Norwegian Government initiated an organized population-based service screening program [10], in which mammography results and interval cancer cases are carefully registered by the Cancer Registry of Norway. The Norwegian Breast Cancer Screening Program (NBCSP) originally included four counties. Other counties were subsequently included, and by 2004 the screening program achieved nationwide coverage. All women between 50 and 69 years of age receive a written invitation biannually, and two-view mammograms from participating women are independently evaluated by two readers.

**Figure 1:**
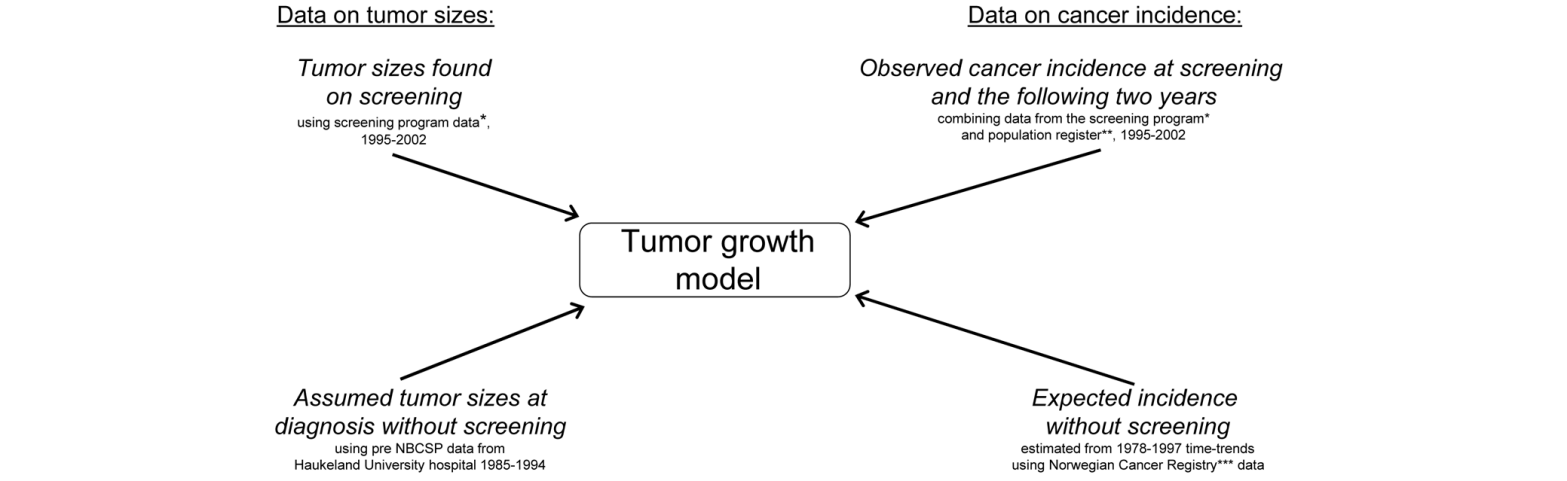
**Data sources used in the estimation**. *Norwegian Breast Cancer Screening Program (NBCSP). **Statistics Norway (SSB). ***Norwegian Cancer Registry.

A high-quality population-based Cancer Registry and a unique personal identity number for each inhabitant in the country enables close follow-up over time [11], and the possibility to link data from several sources (Figure 1). Reporting cancer cases to the Cancer Registry is mandatory, and information is obtained separately from clinicians, pathologists, and death certificates.

**Figure 2:**
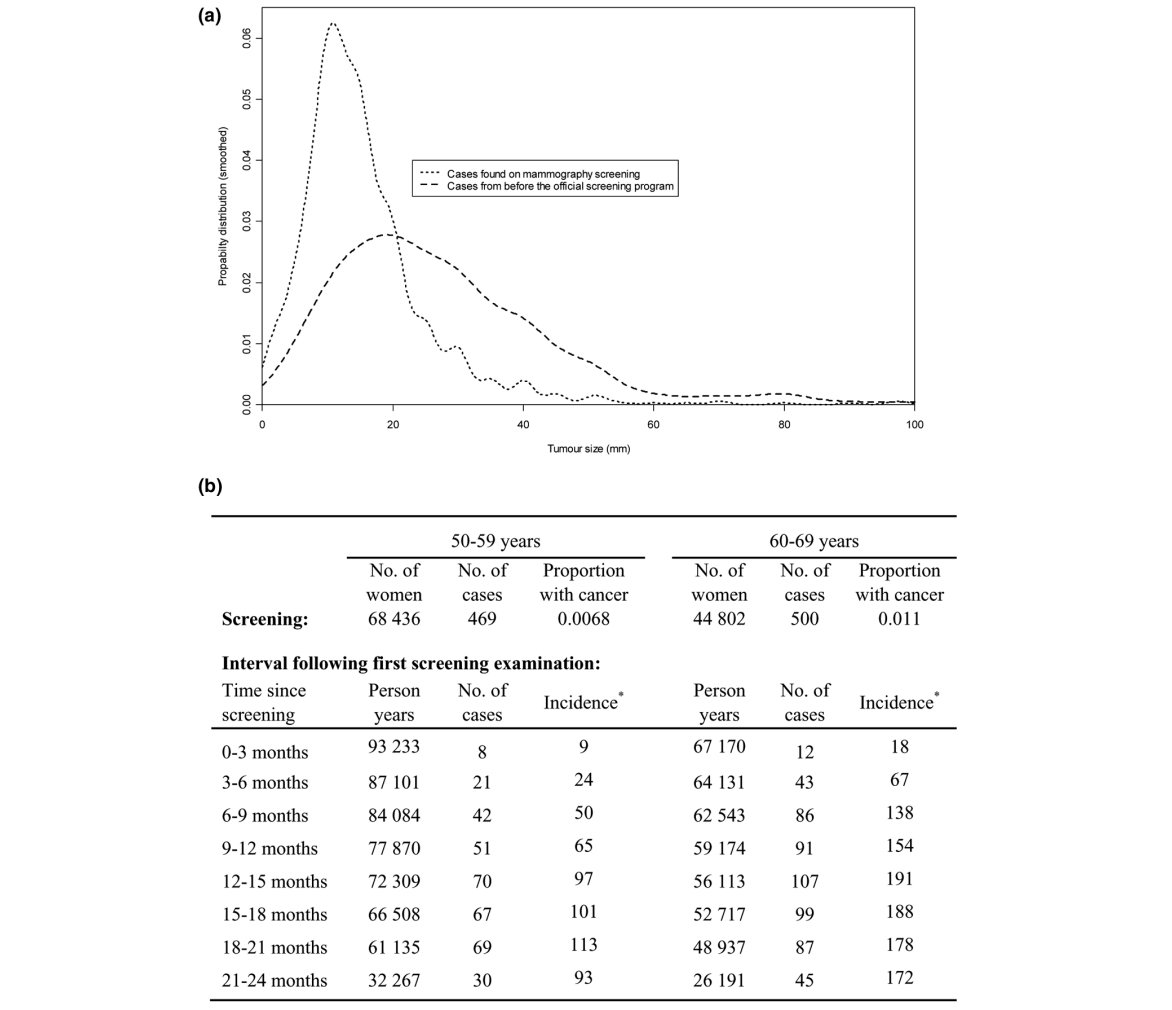
**Summary of data**. **(a) **Distribution of tumor sizes. **(b) **Observed number of cases at first screening and in the following interval. Tumor measurements from before the official screening program started come from a database at Haukeland Hospital (1985 to 1994). Screening data include only the first appearance of women reporting no earlier screening history, while interval data are based on all available observations. *Cases per 100,000 person-years.

The present study includes screening data from 1995 to 2002. A total of 78% of the invited women attended the screening program during this period, resulting in 364,731 screened women 50 to 69 years of age. Among these women, 336,533 answered a question regarding former screening experience – and 113,238 reported no previous (private or public) mammography experience before entering NBCSP. While interval data in this study include the two subsequent years following the first NBCSP attendance of all participating woman, we have chosen to only include screening data from the first NBCSP attendance of women having reported no previous mammography. Eligible women receive a new invitation to mammography screening 16 to 24 months after their previous screening (with most women receiving their invitation 22 to 23 months after the previous screening). All observations are censored 2 days after the new invitation was mailed (or on death, emigration, or after 2 years of observation for women passing the NBCSP upper age limit of 69 years of age). An overview of the data used in the estimation is shown in Figure 2.

To make the results comparable with estimates provided in previous studies [5,12-15], all cases of ductal carcinoma in situ (DCIS) – a noninvasive form of breast tumor – were included. In addition, estimates were also deduced excluding DCIS cases to check the potential effect of DCIS cases. Several tumors detected at the same time in one woman were counted as one case, with size measurements given for the largest tumor. Only new primary breast cancers were included in this study.

In the NBCSP, tumor measurements are performed on pathological sections after surgery, and tumors are measured diagonally between the outer edges. All measurements were performed in a standardized manner according to specifications given in a national quality assurance manual. Tumor size measurements were available for 92% of the cancers detected at screening. There were several reasons for missing tumor measurements: some tumors were torn up at the surgical operation before tumor measurements were taken, others were difficult to measure on pathological cross-sections, and some tumors had grown into the outer skin. In addition, a substantial part had received tumor-reducing treatment before the pathological material was removed. Tumors of unknown size are therefore probably somewhat different from tumors with an observed tumor size. Patients who received tumor-reducing treatment will typically have larger tumors, which in practice could have biased our estimates – leading to higher growth rates. Sensitivity analyses related to possible bias in tumor sizes were therefore performed.

Tumor size measurements of clinical breast cancers that emerge without screening are needed for the tumor growth model suggested in the present article. Since women who do not attend screening represent a selected group, possibly with different alertness to early symptoms, tumor size measurements made before the start of the official screening program were used. The Cancer Registry of Norway did not receive reliable information on tumor size prior to the official screening program. At Haukeland University Hospital (covering Bergen, Norway's second largest city), however, a good database for tumor measurements of clinical invasive breast cancers exists [16]. We were able to use these data, where 503 women aged 50 to 69 years were diagnosed with breast cancer between 1985 and 1994. Among these cases, 433 women (86%) had registered tumor measurements in millimeters. A comparison of tumor measurements found at screening and in the Haukeland University Hospital database of clinically detected cases is shown in Figure 2.

### Growth model specification

**Figure 3:**
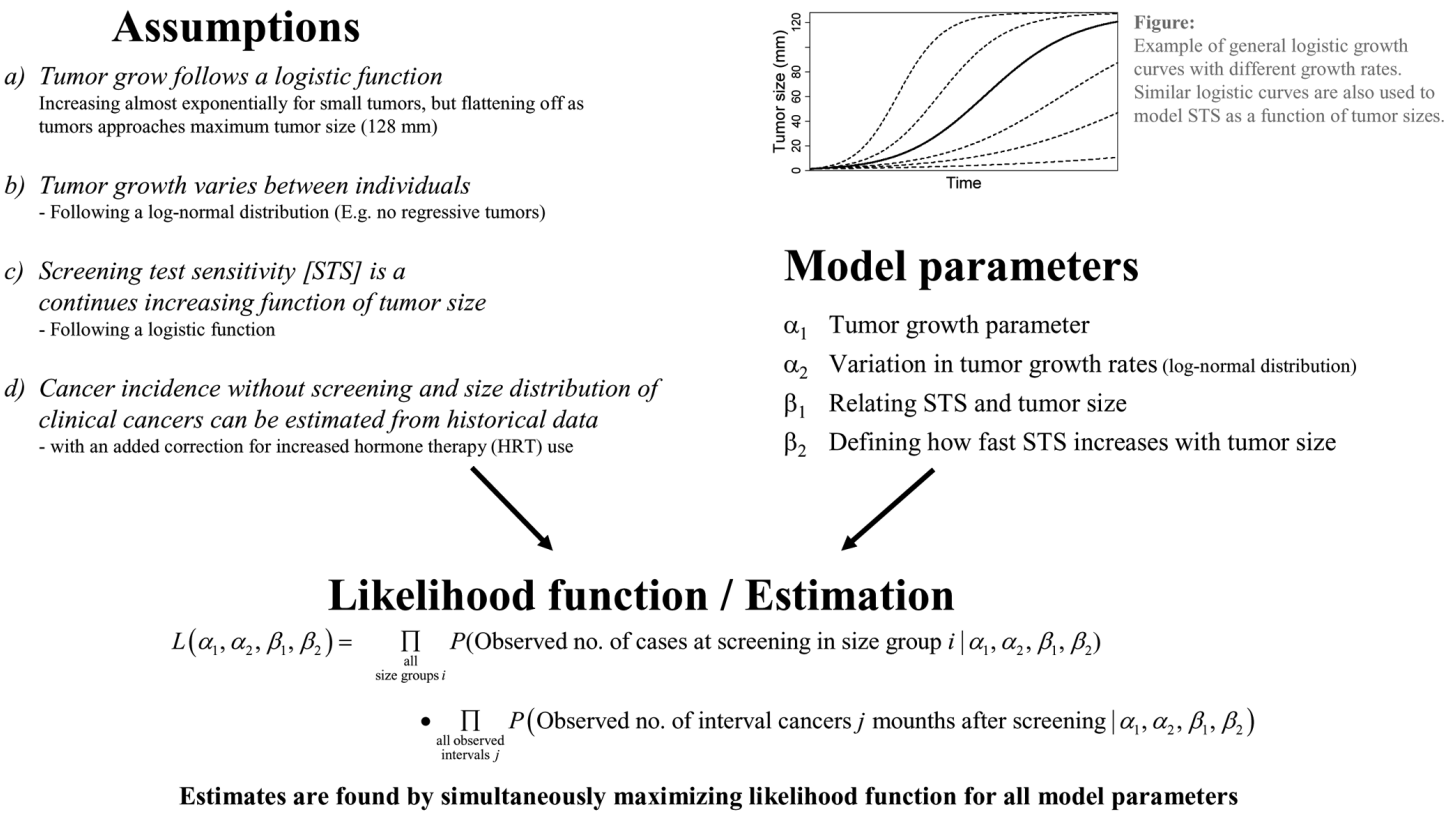
**Overview of the new cancer growth model**. New cancer growth model: assumptions, model parameters, and likelihood function.

Although the growth rates vary throughout the lifespan of each tumor, a smoothly increasing function is likely to serve as a good model for growth rates at the population level, as departures from one individual to the next probably are smoothed out at the population level. For small tumors, growth is mostly governed by the cell reproduction rate of the given tumor cells. This constantly higher growth rate leads to an exponential growth curve with constant doubling times. When tumors grow larger, growth velocity is likely to decrease with the increasing burden on the host, as the tumor receives more limited nutrition. One family of curves starting with near-exponential growth, before gradually leveling off below a given maximum level, is the general logistic function (see examples in Figure 3).

Several studies have examined growth curves, both in general and for human breast cancer tumors in particular. The conclusion has often been that the growth curves can be described by either a logistic function [17] or a Gompertz function [9,18]. For the range of tumor sizes that are relevant for screening, there are only minor differences between logistic and Gompertz growth given probable parameters. Spratt and colleagues used a variant of the general logistic growth curve with a maximum tumor volume of 40 cell doublings, equaling a ball of 128 mm in diameter, after testing several models on a clinical dataset that mostly consisted of overlooked tumors [9,19]. To make the comparison with Spratt and colleagues' observations [9,19], we used the same variant of the log-normal logistic growth model in the present study. This implies an almost exponentional growth for the smallest tumors, with decelerating growth as the tumors approaches the maximum of 128 mm in diameter (see examples in Figure 3). In addition to the chosen model, model fits for several alternative choices of maximum tumor volume were evaluated, with moderate effects on the estimated values.

Growth rates vary between individual tumors, and both a study of overlooked cancers [9] and a thymidine-labeling study of tumors observed in a laboratory [20] found that variations in net productive growth rates (cell production minus cell death) can be described by a log-normal distribution. We therefore modeled the individual growth rates, *κ*_*i*_, by a log-normal distribution with two variables; the mean *α*_1_, and the variance *α*_2_. Mathematically, this gives the following specification of tumor volume, **V_*i*_(*t*)**, as a function of time, ***t***, for a given woman (***i***):

(1) $${\text{V}}_{\text{i}}(\text{t})=\frac{{\text{V}}_{\max}}{{\left[1+\left({\left(\frac{{\text{V}}_{\max}}{{\text{V}}_{\text{cell}}}\right)}^{0.25}-1\right)\cdot e^{-0.25\cdot \kappa_{\text{i}}\cdot \text{t}}\right]}^{4}}$$

where *κ*_*i *_is a log normally distributed growth rate with mean *α*_1 _and variance *α*_2_, V_max _is the maximum tumor volume (set to a tumor of 128 mm in diameter), and V_cell _is the volume of one cell. (As all calculations in the present paper use a relative cancer time, the choice of V_cell _does not affect the given estimates.)

Overall, this can be seen as a mixed effects model with individual logistic growth curves and a log-normally distributed random effect.

Assuming tumors have a ball shape, tumor volumes can be calculated from the tumor diameter, ***X*_*i*_(*t*)**, by:

(2) $${\text{V}}_{\text{i}}(\text{t})=\frac{4}{3}\pi {\left[\frac{{\text{X}}_{\text{i}}(\text{t})}{2}\right]}^{3}$$

As tumor measurements in the NBCSP are the maximum diameter, the real tumor volume will in practice be smaller. The most important part of the model, however, is the modeled growth curve, and sensitivity analyses show little effects of a general reduction in modeled tumor volume as a function of tumor diameter.

### Screening test sensitivity model specification

Since larger tumors are easier to detect on mammograms than smaller tumors, the STS was modeled as an increasing function of the tumor size, *X*, in millimeters. As used for the tumor growth curve, a variant of the logistic function was used for the STS. Mathematically, the modeled STS, *S*(*X*), can be written as:

(3) $$S(X)=\frac{\exp\left(\frac{X-\beta_{2}}{\beta_{1}}\right)}{1+\exp\left(\frac{X-\beta_{2}}{\beta_{1}}\right)}$$

where *β*_1 _defines how fast tumor sensitivity increases by tumor size and *β*_2 _relates STS to tumor size, with *β*_2 _= 0 equaling *S*(0) = 0.5 (places the sensitivity curve in relation to tumor size).

### Parameter estimation

Since mammography screening detects a higher proportion of the larger prevalent tumors compared with the smaller prevalent tumors, the pool of undiagnosed tumors is expected to have a clear overrepresentation of small tumors shortly after screening. One would suspect this could lead to relatively small tumors detected shortly after screening, followed by gradually increasing tumor sizes with the time since last screening. This trend is severely damped, however, as each tumor before detection must reach a certain individual size to produce sufficient symptoms to alarm the woman. In practice, the relationship between tumor size and clinical detection results in only a vague trend in interval cancer tumor sizes by time since screening (correlation = 0.01 in the NBCSP), whereas the number of interval cancers increases sharply. We have therefore chosen to disregard the size distribution of interval cancers, and build our estimation procedure on the observed frequency of interval cancers by the time since screening, the number of cases found at screening, the tumor size distribution of screening cancers, the assumed background incidence, and the size distribution of clinical tumors without screening (based on historical data).

Combining these data with our model, the model parameters can be estimated by maximum likelihood calculation. As the full likelihood includes several integrals, the actual maximum likelihood calculations are performed discretely, grouping the data into sufficiently small time and tumor size intervals.

To ease the calculations, the likelihood contributions from the screening and interval data have been taken as independent. This is possible since the number of cases is small relative to the total population of screened women, and since there probably are considerable variations in tumor growth with several screening detected cancers arising after the observed interval. To test the assumption in a relevant setting, we performed a simulation of the suggested growth model, without the independence assumption, using the estimated parameter values and a 100% overlap in screening and interval populations. This revealed only a weak correlation of 0.019 between the total number of screening and interval cancers (based on 10,000 simulations), giving no indication of problems with the assumed independence. Conditional on the assumed background incidence without screening and the clinical distribution of tumor sizes, the likelihood of a given dataset can be written as:

(4) $$\begin{array}{c} L(\text{data}|\alpha_{1},\alpha_{2},\beta_{1},\beta_{2})\\ \approx P(\text{observed no}\text{. of cases at screening in different size intervals}|\alpha_{1},\alpha_{2},\beta_{1},\beta_{2})\\ \cdot \prod_{\begin{array}{c} \text{all observed}\\ \text{intervals }j \end{array}}P(\text{observed no}\text{. of interval cancers }j\;\text{months after screening}|\alpha_{1},\alpha_{2},\beta_{1},\beta_{2}) \end{array}$$

where the first part is calculated by a multinomial distribution:

(5) $$\begin{array}{l} P(\text{observed no}\text{. of cases at screening in different size groups}|\alpha_{1},\alpha_{2},\beta_{1},\beta_{2})\\ =\frac{sn!}{\prod_{i=1}^{sn}sc_{i}!}\cdot \prod_{i=1}^{sn}sp_{i}^{sc_{i}} \end{array}$$

where *i *is an indicator for the size group, *sn *is the number of screened women, *sc*_*i *_is the number of screening cases in size group *i*, and *sp*_*i *_is the probability of a woman having a tumor in size group *i *at screening, given the parameter set {*α*_1_, *α*_2_, *β*_1_, *β*_2_}.

Similarly, the second part of the likelihood, concerning the rate of interval cancers, follows a Poisson distribution:

(6) $$\begin{array}{l} P(\text{observed no}\text{. of interval cancers }j\;\text{months after screening}|\alpha_{1},\alpha_{2},\beta_{1},\beta_{2})\\ \approx \frac{e^{-ie_{j}}\cdot ie_{j}^{ic_{j}}}{ic_{j}!} \end{array}$$

where *ic*_*j *_is the observed number of cancers *j *months after screening and *ie*_*j *_is the expected number of cancers *j *months after screening, given the parameter set {*α*_1_, *α*_2_, *β*_1_, *β*_2_}.

The probability of finding a cancer in a given size group at screening (*sp*_*i*_) and the expected number of interval cases (*ie*_*j*_), given a set of known parameters, (*α*_1_, *α*_2_, *β*_1_, *β*_2_), are therefore needed for the estimation of model parameters. There is no available knowledge regarding the number of tumors initiated at different ages that have the potential of becoming screening or clinically detected cancers later on. The expected number of cases given a known tumor growth rate cannot therefore be deduced directly. It is possible, however, to calculate the expected number of cases at screening using back calculations from the expected number of clinical cancers seen without screening. This idea is not unlike the theory behind Markov models of cancer screening [12], utilizing known quantities regarding the expected number of future cancers to calculate the expected number of cases at an earlier stage.

Given a set of tumor growth parameters, we can calculate the probability that a tumor arising clinically at a given age without screening would have been in a given tumor size group some months earlier. Combining this with given STS parameters, we can calculate the probability that a tumor arising clinically at a given time without screening is found (earlier) at a given screening examination. Applying this on the expected number of future clinical cancers for all size groups separately, we can calculate the expected number of cancers that would be found at screening and, consequently, the reduction in cancers seen after screening. The probability of finding a given number of cancers in different size groups at screening, and a given number of interval cases each month after screening, can therefore be calculated for a given set of model parameters:

(7) $$sp_{i}=S(\text{Cancer of size }i|\beta_{1},\beta_{2})\sum_{\begin{array}{c} \text{all time}\\ \text{intervals f} \end{array}}r\cdot gs_{f,\text{i}}$$

where *S*(...) is the STS defined in equation (3), and *r *is the expected breast cancer rate per time unit (month) without screening – to simplify calculations, the rate is assumed constant over time as in the earlier used Markov model [5,12], probably giving a good approximation in the limited time span used in the estimation – and *gs*_*f*,*i*_, is the probability that a clinical cancer is in size group *i f *months before clinical detection. Using our assumed tumor growth function, *gs*_*f*,*i *_can be calculated using back calculation of tumor sizes:

(8) $$gs_{f,\text{i}}=\sum_{\begin{array}{c} \text{all}\\ \text{size groups }g \end{array}}p_{g}\cdot P(\text{Tumor of size }g\;\text{was of size }i\;f\;\text{months earlier}|\alpha_{1},\alpha_{2})$$

where *p*_*g *_is the relative proportion of breast cancers of size *g *without screening.

Similarly, *ie*_*j *_can be found by:

(9) $$ie_{j}=PYR_{j}\cdot r\cdot \sum_{\begin{array}{c} \text{all}\\ \text{size groups}\;g \end{array}}p_{g}\cdot fs_{j,g}$$

where *PYR*_*j *_is the number of person years in interval *j *and *fs*_*j*,*g *_is the probability that a clinical cancer in size group *g *would have been found if screened *j *months earlier.

Using back calculation of tumor sizes, *fs*_*j*,*g *_can be expressed as:

(10) $$fs_{j,g}=\sum_{\begin{array}{c} \text{all}\\ \text{size groups}\;gs \end{array}}\begin{array}{c} P(\text{Tumor of size }gs\;\text{was of size g j months earlier}|\alpha_{1},\alpha_{2})\\ \cdot S(gs|\beta_{1},\beta ) \end{array}$$

In practice, both *P*(tumor of size *g *was of size *i f *months earlier |*α*1, *α*_2_) in equation (8) and *P*(tumor of size *gs *was of size *g j *months earlier |*α*1, *α*_2_) in equation (10) can be calculated in the following three stages. First, by rearranging the growth formula equation (1), expressing earlier tumor size as a function of present tumor size and tumor growth rate (*κ*_*i*_). Then calculating upper and lower limits for tumor growth (*κ*_*i*_), constituting the requested probability. Finally, calculating the probability for tumor growth within the given limits using the log-normal distribution and assumed growth parameters {*α*_1_, *α*_2_}.

Combining these formulas, maximum likelihood estimates of the observed dataset can be deduced by numerically maximizing the log-likelihood.

### Modeling choices: specifications

While the number of cancers in the interval between screenings can be observed directly, the expected number without screening has to be estimated. As the NBCSP offers screening to all women in a defined population, no parallel control group is available to carry out this estimation. In addition, commitment to screening can, and probably does, vary with individual risk factors, so those who do not attend are not a suitable control group either.

The background incidence was therefore calculated from historical data combined with an estimated time trend. In practice, data from 1990 to 1994 were used with time trend estimates from an age-period cohort model with additional screening parameters [21]. Incidence rates vary among age groups and counties, and the estimate was therefore weighted by the number of person-years in each combination of age group and county. Further, it may be a problem that the sharply increased use of hormone replacement therapy (HRT) in the 1990s [22] has influenced the historical time trends in breast cancer incidence. HRT is known to increase breast cancer risk [23], and Bakken and colleagues [22] found a relative risk of breast cancer of 2.1 for current versus never users in Norway. Combining sales figures with risk estimates, Bakken and colleagues estimated the proportion of breast cancer cases that could be attributed to HRT use as 27% among Norwegian women 45 to 64 years of age. HRT use increased sharply from the period that was used to calculate the expected incidence without screening (1990 to 1994) to our estimating period (1996 to 2002). Therefore 21% was added to the estimated background incidence (when otherwise not noted), on the basis of information regarding increased breast cancer risk and HRT sale figures found in Bakken and colleagues [22]. With this correction, the expected incidence without screening was estimated as 190 cases/100,000 person-years for women 50 to 59 years of age, and as 219 cases/100,000 person-years for women 60 to 69 years of age.

When calculating the expected number of cases at screening, we cannot include an infinite number of future time intervals. We therefore limited the growth rates to realistic levels given the women's current age, and reweighted the distribution. Experiences with different limits show that the choice of growth limit had little effect on the estimated values.

### Statistical calculations

All calculations, simulations, and plots were performed using the R statistical package [24]. Data were transformed from the Norwegian breast cancer screening database and were summarized using a combination of SQL commands and the statistical package S-PLUS (Insightful, Seattle, USA). To double check the implemented R functions, new datasets were simulated and the results compared with the expected number of cases.

Maximum likelihood estimates were found by optimization over all four parameters simultaneously, using the optimize function found in the R package [24]. For these calculations, time intervals of 1 month were used. Tumor sizes were categorized to 1 mm, 2 mm, 5 mm, 10 mm, 15 mm, ..., 100+ mm, as the background data revealed that many pathologists approximated tumor sizes to the nearest 5 mm, 10 mm, 15 mm, ..., 100 mm (data not shown). To look at possible age differences, estimates were calculated separately for women aged 50 to 59 years and women aged 60 to 69 years, in addition to all age groups combined. Calculations were very computer intensive, with a huge number of probability calculations needed to calculate the expected number of cases for a given parameter set.

The main estimates are presented with (pointwise) confidence intervals showing their (random) uncertainty. Robust 95% confidence intervals were calculated by 1,000 smoothed bias-corrected parametric bootstrap replications [25], resampling all of the observed data except the assumed breast cancer incidence without screening. Simulations were used to deduce the overall STS and the mean sojourn time. As a validation of the model fit, observed values versus expected values were plotted. In addition, the traditional Markov model of breast cancer screening [5,12,26] was compared with the new method using one-fifth holdout cross-validation, measuring the weighted mean square differences. For evaluation of cross-validation results, *P *values calculated from 50 parametric bootstrap replications were used.

## Results

### Parameter estimates

For all age groups combined, model parameters were estimated as {*α*_1_, *α*_2_, *β*_1_, *β*_2_} = {1.07, 1.31, 1.47, 6.51}, while the two age groups 50 to 59 years and 60 to 69 years gave estimates of {1.38, 1.36, 1.50, 6.33} and {0.70, 1.18, 1.46, 6.65}, respectively. While parameters are hard to interpret and compare, several relevant quantities can be deduced once parameters are estimated.

### Estimated tumor growth

**Figure 4:**
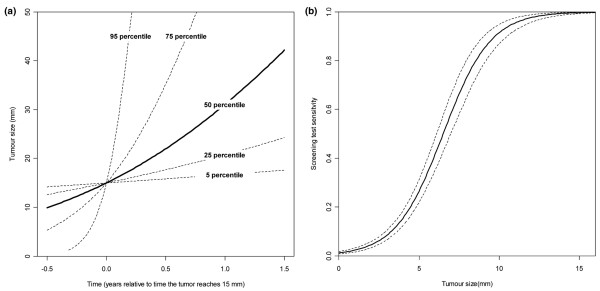
**Estimates of tumor growth rate variation and screening test sensitivity for all age groups combined**. Estimates for all age groups combined, with correction of background incidence (+21%) due to increased hormone therapy use. **(a) **Estimated variation of tumor growth rates, illustrated by growth curves for the 5th, 25th, 50th, 75th and 95th percentiles. **(b) **Estimated screening test sensitivity with 95% pointwise confidence intervals.

The estimated tumor growth implies that tumors in women 50 to 59 years of age take a mean 1.4 years to grow from 10 mm to 20 mm in diameter, while tumors in women 60 to 69 years of age take a mean time of 2.1 years (Table 1). Overall, the mean time taken to grow from 10 mm to 20 mm was estimated as 1.7 years, but there were large individual variations with an estimated standard deviation of 2.2 years. If we removed the correction for a probable higher background incidence due to increased HRT use, growth rates were somewhat lower (Table 1). There were generally large variations in tumor growth (Figure 4a), and tumor-doubling times at 15 mm varied from 41 days for the first quartile to 234 for the last quartile (Table 1). Comparing the new estimates with earlier estimates based on overlooked cancers found in Spratt and colleagues [9] we found generally good concordance, with only slightly more very fast-growing tumors (Table 2).

**Table 1:** Summary of results with 95% bias-corrected bootstrap confidence intervals

	With supposed higher background incidence due to increased hormone therapy use (+21%)			Combined estimate (50 to 69 years) with non-adjusted background incidence
		
	Women aged 50 to 59 years	Women aged 60 to 69 years	Combined estimate (50 to 69 years)	
Time taken from 10 mm to 20 mm (years)				
Mean	1.4 (1.1, 1.5)	2.1 (1.8, 2.4)	1.7 (1.5, 1.8)	2.0 (1.8, 2.2)
Standard deviation	1.9 (1.7, 2.2)	2.4 (2.1, 2.7)	2.2 (2.0, 2.4)	2.7 (2.5, 2.9)
Volume doubling time at 15 mm (days)				
25th percentile	29 (19, 36)	65 (47, 79)	41 (32, 48)	29 (21, 35)
50th percentile	73 (56, 86)	143 (116, 165)	99 (84, 111)	94 (77, 107)
75th percentile	180 (148, 205)	308 (253, 352)	234 (204, 259)	287 (243, 322)
Screening test sensitivity				
5 mm	29 (21, 36)	24 (18, 30)	26 (22, 31)	26 (22, 31)
10 mm	92 (88, 99)	91 (86, 98)	91 (88, 96)	91 (87, 96)
Indicators of potential screening efficacy				
Mean sojourn time (years)	2.3 (2.0, 2.6)	3.5 (3.1, 3.9)	2.8 (2.6, 3.1)	3.4 (3.1, 3.6)
Proportion of tumors visible on screening	0.95 (0.94, 0.96)	0.95 (0.95, 0.96)	0.95 (0.95, 0.96)	0.95 (0.95, 0.96)

**Table 2:** Estimates of tumor growth rates compared with Spratt and colleagues' [9] estimates based on overlooked and nontreated cancers

Percentile	Growth parameter (*κ*_*i *_in equation (1))		Time (years) tumor takes to grow from 10 mm to 20 mm	
	
	Estimate	Spratt et al.	Estimate	Spratt et al.
1st	0.2	0.2	10.9	12.9
5th	0.4	0.6	6.3	4.6
25th	1.3	1.7	2.0	1.5
50th	3.0	3.2	0.9	0.8
75th	7.2	5.2	0.4	0.5
95th	25.4	11.8	0.1	0.2
99th	61.9	33.7	0.0	0.1

### Estimated screening test sensitivity

The mammography STS was estimated to increase sharply from around 2 mm to 12 mm, with the STS reaching 26% at 5 mm and 91% at 10 mm (Figure 4b). There was no significant difference in the estimated STS between the two age groups (*P *= 0.83 for the STS at 5 mm).

### Overall screening test sensitivity and mean sojourn time

Using simulations to combine the STS and the given distribution of clinical tumors, we found that nearly all cancers were likely to be visible at screening before reaching clinical detection (Table 1). Defining the mean sojourn time as the time tumors are visible at screening before clinical detection, these cancers have a mean sojourn time of 3.0 years – resulting in an overall mean sojourn time of 2.9 years for all cases. In older women the mean sojourn time was estimated to be significantly longer. There were large variations in the sojourn time, and the standard deviation was estimated as 5.0 years, indicating that the Markov model (which equals the mean sojourn time and standard deviation) does not allow for enough individual variation in growth rates.

### Model fit

**Figure 5:**
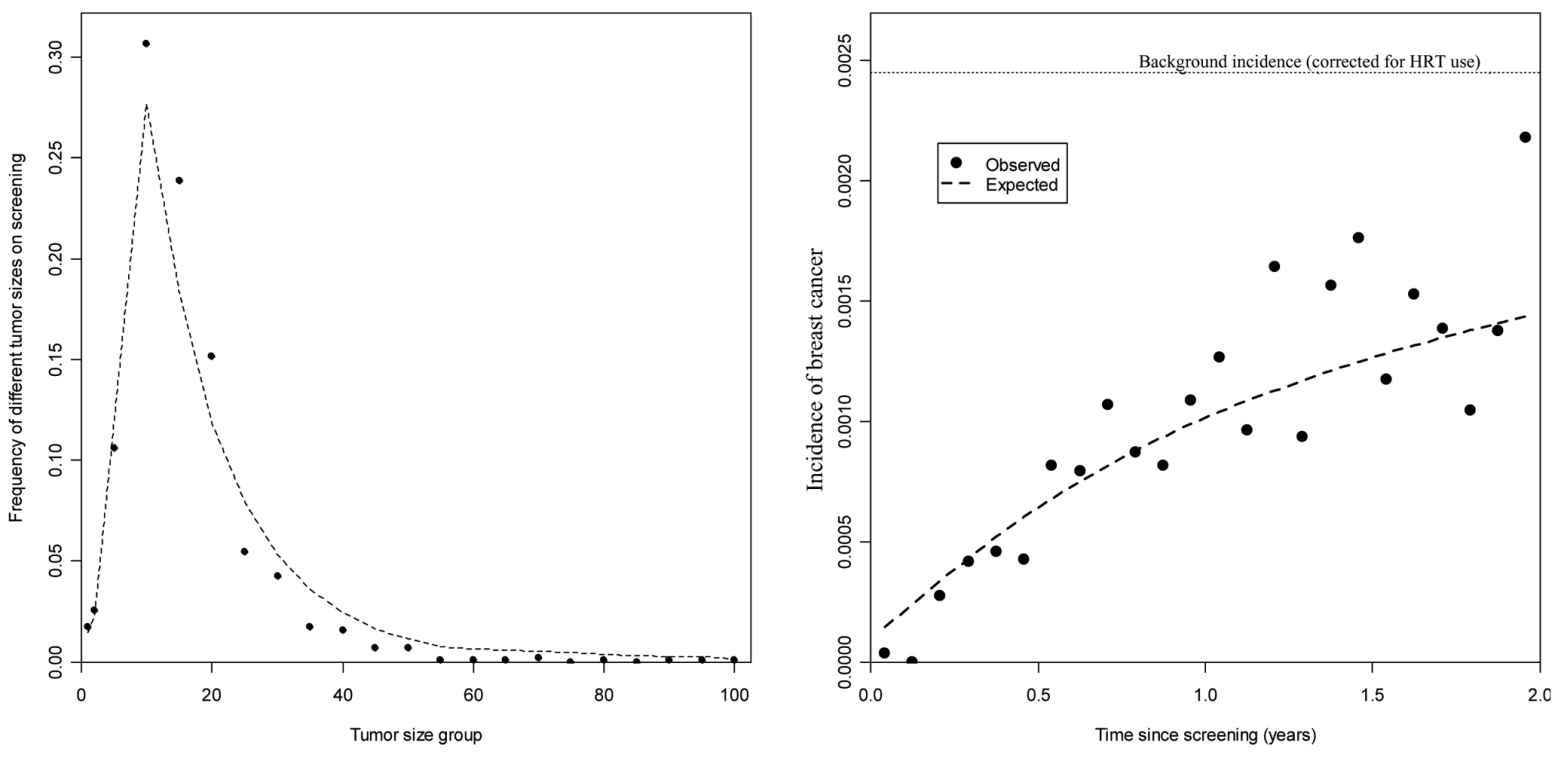
**Model fit using the new cancer growth model**. **(Left) **Tumor sizes on screening. **(Right) **Number of interval cancers. HRT = hormone replacement therapy.

The overall model fit was very good (Figure 5). Comparing the model fit by looking at the number of cancers at screening and the following interval, the new model gave significantly (bootstrap *P *< 0.0001) better model fit than the classical Markov model [26]. Overall, the predictive power increased by 85% (that is, an 85% reduced weighted difference between observed and predicted values, when evaluated through cross-validation).

A more exponential tumor growth curve modeled through a higher maximum tumor volume weakened the overall model fit (data not shown), supporting the assumption that the doubling time of the tumor volume may increase with increasing tumor size (as assumed by the logistic model). To explore possible biases due to missing tumor measurements at screening, we applied several different assumptions regarding the true tumor diameter of the unknown tumors, revealing very stable parameter estimates (data not shown).

## Discussion

The present study introduces a new way of modeling cancer growth and STS, based on data from a large screening program. Tumor growth was estimated to vary greatly between individual tumors, with tumors taking a mean time of 1.7 years to grow from 10 mm to 20 mm in diameter. The STS was estimated to increase rapidly with tumor size, from 26% at 5 mm to 91% at 10 mm.

Applied to the NBCSP data, the new model gives a very good model fit, and a significantly better predictive power than the previously used Markov model [26]. Certain aspects of the model need further investigation, however, and some have argued that cancer growth either follows exponential [27] or Gompertz [18] growth functions, and not the assumed logistic growth curve [19]. The practical difference between the logistic and Gompertz curve is relatively small, but an exponential growth curve could probably alter the results significantly. Mathematically, a logistic function with very large maximum tumor volume almost equals the exponential curve. Several alternative levels of maximum tumor volume were therefore tested, giving weaker model fit as the maximum tumor volumes increased, thereby strengthening our assumption of a bounded growth function (rather than an exponential growth function).

Another possible objection to the model is that the STS is assumed to always increase towards 100% as the tumor size increases, while some cancers probably never become visible on mammograms [28]. To test this alternative hypothesis, a three-parameter STS function with a parameter for maximum STS was tested, giving no indication of a lower maximum STS level. To limit the complexity of the estimation procedure and the presentation of the new model, only data from the first screening round were used in this study. Data from subsequent screening rounds were still available, and while the model predicted a 71% decline in detected cancers from first screening to second screening, the observed decline was only 46%. This is a considerable predicted–observed difference, and the NBCSP generally has shown a surprisingly high cancer rate at the second screening. In addition to possible problems with the model itself, this can be an effect of changes in HRT use in the study period (increasing the general breast cancer risk), of increased sensitivity in the second round due to use of earlier mammograms, of better training of staff with time, or of an overrepresentation of communities with high cancer risk in the second screening round.

Even with a high-quality cancer registry, problems with the applied data may cause more bias to the estimated values than the applied model assumptions. Studying the fit of the new model (Figure 5), there are some signs of discrepancy in the last half of the interval following screening, with too many observed cases. This may be an effect of unregistered opportunistic screening, since opportunistic screening has been available at many private institutions, and cancers detected outside the NBCSP have in practice been registered as interval cancers. Unfortunately, no detailed information is available on the extent of opportunistic screening in the different age groups, and there is no precise information on whether interval cancers have been detected by opportunistic screening or clinical symptoms. Preliminary studies by the Norwegian Cancer Registry indicate that approximately 10% of the NBCSP's invited women are screened outside the program each year. This percentage may, however, be lower since the level of opportunistic screening may be higher among nonattendees of the public screening. Preliminary attempts to estimate the level of opportunistic screening, and to correct the estimated STS and growth rates, indicate little bias in the estimated mean cancer growth and the STS, while the variation in cancer growth rates decreased substantially.

Another problem can be the assumed background incidence without screening, as the estimates changed somewhat (Table 2) when removing the correction for a probable higher background incidence due to increased use of HRT [22]. The correction probably improves the estimates, but there is uncertainty. Based on typical user patterns, it is possible that HRT use could have been higher than assumed among woman 50 to 59 years of age, and somewhat lower among the 60 to 69 years age group. The correction may therefore be too small for the younger age group and too strong for the older age group. In addition, HRT use fluctuated during the study period, and may have influenced the cancer incidence, the STS and the tumor growth in different ways. Most importantly, HRT use is known to reduce the STS [29-31], at least partially due to increased breast density. Since HRT use has been quite common in Norway, the STS may have been even higher with moderate levels of HRT use. Tumor growth estimates may also be affected by HRT use, and both the STS and tumor growth estimates should be viewed in the light of the relatively high HRT use during the study period.

Overall, sensitivity investigations indicate that the new model is probably less vulnerable to several potential biases than the Markov model [5,12], possibly as a result of more utilized data. The model is substantially different from the Markov model, rendering direct comparisons difficult, but the slightly different overall screening efficiency indicators confirm the estimated mean sojourn time and STS from other studies [32,33], with a shorter mean sojourn time and a higher STS than found in a recent Norwegian study [26].

More importantly, the new model estimates tumor growth directly connected to tumor measurements, similar to the earlier nonpopulation-based studies of overlooked cancers [4,9], but using a much larger population-based material. The results confirm previously reported growth rates (Table 2), large variances in tumor growth, and a probable bounded growth function, suggesting less selection bias in studies of (earlier) overlooked cancers than previously assumed [9].

Earlier studies have shown decreasing tumor progression and higher STS with increasing age [32,33]. The present study confirms the previously reported decrease in tumor growth with age, but we found no trend in STS associated with age. This is surprising, but very few new breast cancers were diagnosed in the first months after screening among women 50 to 59 years of age (Figure 1), indicating a surprisingly high STS for the younger age group. An investigation of which aspects of the data influence the various parameters revealed that differences in tumor size between screening and clinically detected tumors are vital for STS estimates. In the Norwegian screening program there is little difference in screening and clinical tumor sizes between the two age groups, a fact that indicates small differences in STS by age. This could of course be an artifact due to the modeling, but could also be an effect of very different recall rates in the two age groups [34]. Indeed, the issue clearly motivates further examinations of the STS among younger women.

Compared with studies of overlooked cancers and with studies of women who refused treatment, the population-based approach greatly increases the number of observed cases and applies data that are probably less biased. Generally, this model combines many of the advantages of the large population-based Markov methods [5,12], with more specific tumor growth estimates found in clinical studies of overlooked cancers. This makes the model suited for both optimizing screening designs and cost–benefit analyses.

By combining the present tumor growth and STS estimates with death rates, different screening designs may be evaluated even more efficiently than seen in earlier studies. Plevritis presented an advanced simulation approach with continuous tumor growth [35]. The approach was based on similar tumor size back-calculation techniques to those used in our study, but the lack of estimates probably limited the practical impact of that study. In practice, Markov models have often been used to evaluate screening designs [36], but without a direct link to tumor size it is difficult to separate and compare the mean sojourn time and the STS between screening programs [26]. In recent years, more advanced simulation models have been suggested – as seen in the US National Cancer Institute Cancer Intervention and Surveillance Modeling Network [1,37] – further emphasizing the need for precise tumor growth estimates.

**Figure 6:**
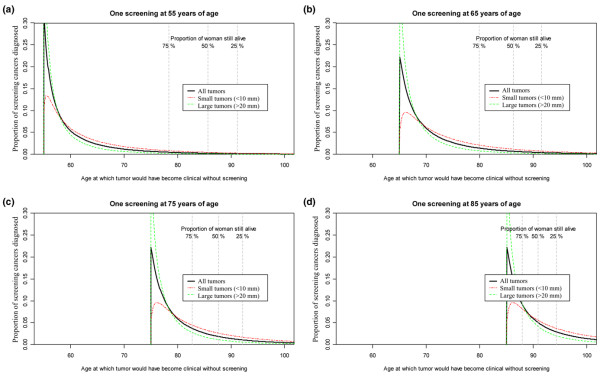
**Illustration of potential use of the new cancer growth model**. Age at which screening tumors would have become clinical without screening, by tumor size at the time of screening detection. **(a) **Screening at 55 years of age. **(b) **Screening at 65 years of age. **(c) **Screening at 75 years of age. **(d) **Screening at 85 years of age. Vertical lines mark the expected time at which 25%, 50% and 75% of the screened women are suspected to have died, based on death rates from Statistics Norway. Panel (c) and (d) are based on the screening test sensitivity and growth estimates from the 60 to 69 years age group.

Whereas screening with mammography has been related to reduced mortality in several randomized trials [32,38], so-called overdiagnosis remains a controversial topic. Following the conservative definition of the number of overdiagnosed cases as 'the number of women who would not had breast cancer in their life time without participating in mammography screening', our new model can be used to estimate the level of overdiagnosis under different screening designs. As a motivation for further studies, we have estimated the probable age at which screening-detected cancers would have become clinically detected without screening, given one screening examination at different ages. Figure 6 illustrates why screening in higher age groups is controversial, since a large proportion of cancers would never have surfaced in the absence of screening. On the other hand, our estimates indicate that the vast majority of screening cancers in the current NBCSP age group (50 to 69 years) would at one stage been detected clinically without screening. The new method presented here provides a toolbox for estimating this and other central issues related to mammography screening.

Although the new model may be closer to the underlying biological process than the Markov model [5,12], there is a potential for improvements of the model. For example, the model assumes that tumors do not regress but in the literature there are a few reports of regressive breast cancers [39], and it is possible that a certain proportion of cancers stop growing or regress. This may particularly apply to noninvasive disease. To test the vulnerability of this possibility in our estimates, we calculated the estimates excluding DCIS cases – assuming that all DCIS cases regress, showing very little effect on estimated values (Table 3). Still there could be a significant proportion of DCIS cases that do regress, with great relevance for DCIS treatment. Hence, an expansion of the model could, for example, be to add a separate parameter for regressive DCIS.

**Table 3:** Estimates with and without ductal carcinoma *in situ* (DCIS)

Data used	Time taken (years) to grow from 10 mm to 20 mm		Volume doubling time (days) at 15 mm			Screening test sensitivity		Indicators of potential screening efficacy	
	
	Mean	Standard deviation	25th percentile	50th percentile	75th percentile	5 mm	10 mm	Mean sojourn time (years)	Proportion of tumors visible on screening
With ductal carcinoma *in situ*	1.7	2.2	41	99	234	26	91	2.9	0.95
Without ductal carcinoma *in situ*	1.5	1.9	44	96	209	23	91	2.5	0.95

All other estimates in the present article are given including ductal carcinoma *in situ*.

## Conclusion

To summarize, tumor growth and STS estimates can be directly linked to tumor size in a full population study, resulting in very useful growth estimates directly connected to a biologically relevant measure. Tumor growth seems to vary greatly between tumors, with higher growth rates among younger women. Most tumors become visible at screening when they reach a diameter of 5 mm to 10 mm.

## Abbreviations

DCIS = ductal carcinoma *in situ*; HRT = hormone replacement therapy; NBCSP = Norwegian Breast Cancer Screening Program; STS = screening test sensitivity.

## Competing interests

The authors declare that they have no competing interests.

## Authors' contributions

HW-F proposed the article and method, collected data from the screening database, and performed the statistical analyses and programming. The author's main supervisor ST together with the other coauthors participated in initial project meetings and guided the candidate through the process. HW-F drafted the paper, receiving substantial assistance from LJV, ST, BHL and OOA with the written presentation.
